# Abiotic and biotic responses to woody debris additions in restored old fields in a multi‐site Before‐After‐Control‐Impact experiment

**DOI:** 10.1002/ece3.9058

**Published:** 2022-07-04

**Authors:** Tina Parkhurst, Suzanne M. Prober, Mark Farrell, Rachel J. Standish

**Affiliations:** ^1^ Harry Butler Institute Murdoch University Murdoch Western Australia Australia; ^2^ CSIRO Land and Water Wembley Western Australia Australia; ^3^ School of Biological Sciences The University of Western Australia Crawley Western Australia Australia; ^4^ CSIRO Agriculture and Food Glen Osmond Western Australia Australia

**Keywords:** Formicidae, herbaceous vegetation, MBACI design, old field restoration, soil carbon, soil moisture, soil organic matter, woody debris

## Abstract

Ecological restoration of former agricultural land can improve soil conditions, recover native vegetation, and provide fauna habitat. However, restoration benefits are often associated with time lags, as many attributes, such as leaf litter and coarse woody debris, need time to accumulate. Here, we experimentally tested whether adding mulch and logs to restoration sites in semi‐arid Western Australia can accelerate restoration benefits. All sites had been cropped and then planted with native trees and shrubs (i.e., *Eucalyptus*, *Melaleuca*, and *Acacia* spp.) 10 years prior to our experiment, to re‐establish the original temperate eucalypt woodland vegetation community. We used a Multi‐site Before‐After‐Control‐Impact (MBACI) design to test the effects on 30 abiotic and biotic response variables over a period of 2 years. Of the 30 response variables, a significant effect was found for just four variables: volumetric water content, decomposition, native herbaceous species cover and species richness of disturbance specialist ants. Mulch addition had a positive effect on soil moisture when compared to controls but suppressed growth of native (but not exotic) herbaceous plants. On plots with log additions, decomposition rates decreased, and species richness of disturbance specialist ants increased. However, we found no effect on total species richness and abundance of other ant species groups. The benefit of mulch to soil moisture was offset by its disbenefit to native herbs in our study. Given time, logs may also provide habitat for ant species that prefer concealed habitats. Indeed, benefits to other soil biophysical properties, vegetation, and ant fauna may require longer time frames to be detected. Further research is needed to determine whether the type, quantity, and context of mulch and log additions may improve their utility for old field restoration and whether effects on native herbs are correlated with idiosyncratic climatic conditions.

## Graphical abstract

Benefits of ecological restoration are often associated with time lags, as many attributes, such as leaf litter and coarse woody debris, need time to accumulate, particularly in arid ecosystems. Here we used a 2 year MBACI experiment to test if adding woody debris to restored eucalypt woodlands in semi‐arid western Australia can accelerate restoration outcomes. We found that only few abiotic and biotic variables responded positively, and conclude that detectable benefits of additional restoration actions may be slow in arid ecosystems.

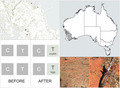

## 1 INTRODUCTION

Agricultural land practices can lead to land degradation and biodiversity loss. Ecological restoration of abandoned agricultural land is a key activity to improve biodiversity and ecosystem functioning. In particular, active restoration measures (i.e., direct seeding and seedling planting) in agricultural landscapes where abiotic and biotic barriers persist (Cramer et al., 2008) have the potential to improve soil condition and habitat suitability for fauna. However, full ecosystem recovery may not occur, even after long timeframes (i.e., decades to centuries) (Isbell et al., 2019; Parkhurst, Prober, Hobbs, & Standish, 2021).

Incomplete recovery of biodiversity and ecosystem functions on restored old fields may be due to abiotic and biotic constraints to recovery, such as depleted soil chemical and biophysical functions, altered edaphic properties, and competition mechanism of native and invasive plant species (Shackelford et al., 2021) (Flinn & Marks, 2007; Piché & Kelting, 2015; Standish et al., 2006). For example, compacted soils and depleted soil carbon concentrations limit key ecosystem functions such as water infiltration and water storage capacity, therefore reducing ecosystem productivity (Franzluebbers, 2002).

In addition, recovery may also be limited by time lags in the development and repair of fauna habitat and ecosystem functions (Isbell et al., 2019; Prober et al., 2014; Vesk et al., 2008). In particular, resources such as leaf litter, and fine and coarse woody debris in young restoration sites, are less abundant than in mature vegetation states (Manning et al., 2013; Parkhurst, Prober, & Standish, 2021). Yet, leaf litter and woody debris are vital components of the plant–soil feedback (Sayer, 2006) and provide essential resources and important habitat components to fauna (Gibb & Cunningham, 2013; Sandström et al., 2019; Sayer et al., 2006).

Debris from the planted vegetation interacts directly and indirectly with the soil surface's physical and biogeochemical functions through complex processes and feedback loops (see Figure 1 in Prober et al., 2014). Direct interactions include not only diverting water run‐off and providing a protective surface layer that reduces evaporation and loss of soil moisture, soil surface temperatures, erosion, and mineral leaching but also presents a physical barrier for seeds and seedlings (Bowman & Facelli, 2013; Lindenmayer et al., 2002; Xu et al., 2013). These changes to water and temperature facilitate further changes, such as increased soil organic matter and biological activity, which in turn, result in altered soil surface properties (i.e., reduced compaction), soil structure and texture, and carbon and nutrient cycling (Colloff et al., 2010; Sayer, 2006). Improvements in soil physical and chemical conditions can then positively influence plant establishment and growth, stimulate soil microbiological activity and alter decomposition rates, and promote soil‐ and surface‐active invertebrates, creating a feedback loop to ecosystem functioning (Colloff et al., 2010; Sayer et al., 2006; Snyder & Hendrix, 2008).

**FIGURE 1:**
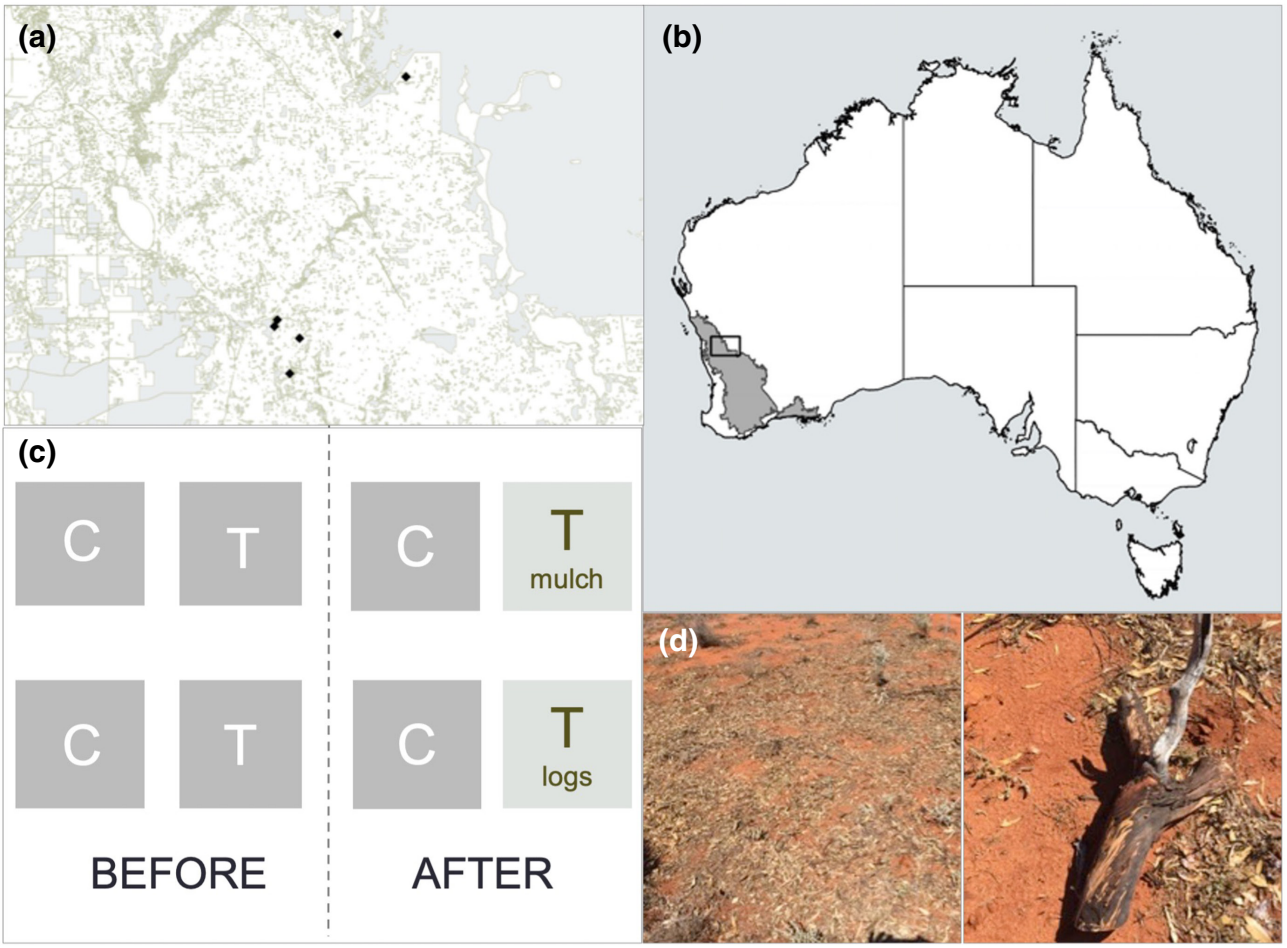
(a) Study location extent in the western Australian wheatbelt; (b) five experimental study sites; (c) four MBACI treatment plots per study site; and (d) mulch and log addition treatment application in 2017

Litter and woody debris also directly shape plant species composition by either promoting or suppressing seedling germination, emergence, and survival in patches where it is present (Bowman & Facelli, 2013; Facelli & Pickett, 1991). In particular, plant species diversity (Xiong & Nilsson, 1999) and understory vegetation patterns are influenced by leaf litter inputs and deposition patterns (Sydes & Grime, 1981).

For ground‐dwelling and soil invertebrate fauna, litter and woody debris provide habitat (Sandström et al., 2019; Seibold et al., 2015). In particular, litter, but also fine and coarse woody debris, maintain a stable microclimate by reducing fluctuations of soil moisture content and soil surface temperatures (Fekete et al., 2016). In addition, litter and woody debris provide habitat through the provision of food sources, nesting sites, and refugia from climatic conditions and predators (Gibb et al., 2006, 2012; Sayer, 2006).

Adding fine and coarse woody debris either for the purpose of soil amendments or habitat improvements has been found beneficial for restoring soil conditions and fauna habitat at restored mine sites (Adl, 2008; Craig et al., 2014), soil biophysical properties, understory plants (Goldin & Hutchinson, 2015; Prober et al., 2014), and habitat for reptiles and birds in degraded and restored temperate woodlands (Mac Nally, 2006; Mac Nally et al., 2001; Manning et al., 2013; Shoo et al., 2014).

However, understanding of how additional restoration measures may accelerate desired changes to soil chemical and biophysical functions, native herbaceous vegetation, and invertebrates after old field restoration in semi‐arid landscapes is limited (Sandström et al., 2019). This is despite the urgent need to improve restoration outcomes in agricultural landscapes across the globe (Parkhurst, Prober, Hobbs, & Standish, 2021). Arid to semi‐arid landscapes require particular attention because low and variable rainfall patterns, as well as slow biomass production, can prolong ecosystem recovery following restoration actions (Aronson et al., 1993).

In this study, we experimentally tested whether the addition of mulch and wood (proxies for leaf litter and fine debris, and coarse woody debris, respectively) accelerates restoration outcomes after 2 years in young (~10 years) restoration plantings in a semi‐arid agricultural landscape in Western Australia. Old fields had been planted with native woody vegetation aiming to restore the native reference eucalypt woodland community.

To measure the effectiveness of our restoration treatments, we drew on key measures of soils, vegetation, and fauna communities to provide a broad representation of biodiversity and ecosystem processes known to be valued or important for ecosystem functions. For soils, we focused on biogeophysical measures as those are key functional restoration barriers and are often understudied (Kollmann et al., 2016). For vegetation, the herbaceous layer is particularly vulnerable to degradation and weed invasion, yet supports about half of the diversity in these ecosystems (Parkhurst, Prober, & Standish, 2021), hence is a critical restoration focus. For fauna, we focused on ants because they are responsive to old field restoration interventions and may indicate impending recovery of other fauna (Parkhurst, Standish, Andersen, & Prober, 2021; Sandström et al., 2019). Ants have been widely used as bioindicators of ecological change, both at the species and functional group level (Andersen & Majer, 2004; Hoffmann & Andersen, 2003; King et al., 1998). Here, we focus on functional groups based on their habitat preferences and adaptations to environmental stressors as proposed by Andersen (1995) because they can provide important insights into the restoration process. In particular, key ant functional groups (e.g., cryptic species, subordinate Camponotini, hot and climate specialists) show responses to land conversion in temperate zones (de Jesus Santos et al., 2021).

We used a multi‐site before‐after‐control‐impact (MBACI) experimental design to examine the effects of woody debris addition on soil condition and biodiversity (flora and ants) to accelerate the restoration of old fields (Green, 1979; Underwood, 1994).

We hypothesized that the addition of mulch and logs to restored old fields in a semi‐arid agricultural landscape would:

1. improve soil biophysical condition, specifically increase soil moisture, soil organic matter and carbon, and available nitrogen (Prober et al., 2014; Sayer, 2006), therefore reduce bulk density, increase soil microbial activity, and decomposition rates (Xu et al., 2013), (*n* = 14 response variables).
2. reduce bare ground and increase woody debris, as well as increase herbaceous vegetation cover and richness due to improved soil condition, while potentially suppressing native herbaceous species, which are predominantly fine seeded (Prober et al., 2014), (*n* = 6 response variables).
3. provide habitat for ants, evidenced by increased abundance and diversity of functional groups that forage and nest in woody debris (e.g., cryptic species) (Gibb & Cunningham, 2013), and reduce abundance and richness of ant species with a preference for hot, open areas (e.g., hot climate specialist) (Hoffmann & Andersen, 2003) (*n* = 10 response variables).

## 2 MATERIALS AND METHODS

### 2.1 Study sites

Experimental sites were established in the northern wheat‐growing district of Western Australia (Lat −29.66°, Long 116.18°) in August 2017, and monitored through to November 2019. The landscape is dominated by agriculture (grazing and cropping), and remnants of native vegetation are small and highly fragmented (Figure 1). A Mediterranean to semi‐arid climate, with dominant but variable winter rainfall characterizes the region (Hobbs, 1993). During the study, winter rainfall was dominant but bolstered by significant, unusual spring and summer rainfall in 2017 (Figure S1). Rainfall varied spatially too. The two northern sites received 198.9 mm of rain in 2017 and 181.9 mm in 2019, well below the long‐term annual mean of 325 mm (recorded at the nearest rainfall station in the town of Perenjori [Bureau of Meterology, 2020]). Rainfall for the three southern sites totaled 371 mm in 2017 and 215 mm in 2019. The long‐term annual average is 334 mm (recorded at the nearest rainfall station on Koobabbie farm near the town of Coorow).

We selected five planted old field sites with similar soil types and vegetation composition. Old fields were planted with York gum (*Eucalyptus loxophleba* Benth.) and dominant shrubs as understory (planting and species details provided in Parkhurst, Prober, & Standish, 2021). At the time of sampling in 2017, vegetation age ranged from 8 to 13 years and the distance from remnant measured 279 m (±162 m).

### 2.2 Experimental design

We established two control and two treatment plots, each measuring 5 m × 5 m, in the interrows of five planted old field sites (Figure 1). Both treatments were randomly assigned to plots within each site. Between August and early November 2017, we measured a total of 30 response variables at each of the control and treatment plots (Table S1). Response variables included soil physical and chemical properties (bulk density, penetration resistance, soil moisture, and nitrogen and carbon pools), microbial biomass, decomposition rate of rooibos and green tea as per the standardized Tea Bag Index (TBI) protocol developed for comparison of litter decomposition rates across various ecosystems by Keuskamp et al. (2013), herbaceous vegetation cover and richness, and ant abundance and richness, as well as abundance and richness of ant functional groups (Table S1). Detailed sampling method descriptions are provided in the Supporting Information section.

In November 2017, one treatment plot at each site was uniformly covered with 13 kg of freshly mulched York gum branches including leaves, and a second treatment plot with three York gum logs (average length and circumference = 80.3 cm [1–121 cm] and 33.2 cm [13–62 cm]) (Figure 1). The mulch and log application rate mimics leaf litter and fine and coarse woody debris cover of the intact York gum woodland remnants as presented in Parkhurst, Prober, and Standish (2021). York gum mulch was sourced from roadside tree lopping of a local shire and the logs were cut to size from recently fallen York gum branches.

**FIGURE 2:**
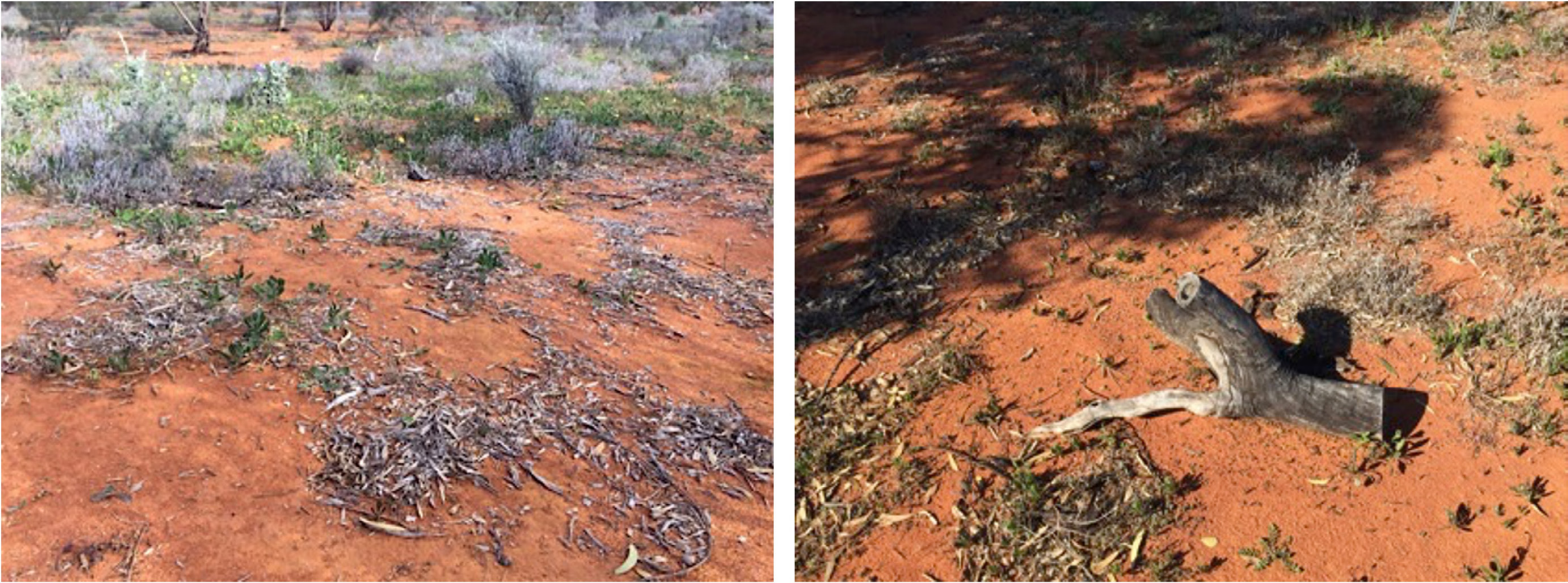
Example mulch and log addition treatments after 2 years showing patchy mulch distribution and some aging of the log surface

After 2 years, between August and November 2019, we re‐measured all 30 response variables across the control and treatment plots (Figure 2, Table S1).

### 2.3 Data analysis

We used a multi‐site before‐after‐control‐impact (MBACI) design (Underwood, 1991) to evaluate changes resulting from log and mulch additions at planted old field sites on soil chemical and biophysical properties, and vegetation and ant communities. The multi‐site BACI design was chosen to increase the reliability of detecting a treatment effect because it controls for non‐treatment variation (Underwood, 1994). In addition, the MBACI design is suitable in landscapes such as ours where ecological variation due to climate and other factors is high but decipherable by comparing BACI plots. The analysis of the Before‐After‐Control‐Impact experiment tests for a significant interaction term because this signifies a bigger effect of the treatment than time. In other words, the analysis detects an impact when the change in the BA factor is significantly different for the impact samples compared to the control samples (i.e., difference of the mean of the two changes [control_after_ − control_before_] − [impact_after_ − impact_before_]).

To determine the potential effects of log and mulch additions on soil chemical and biophysical properties, and vegetation and ant communities, we ran linear mixed models using the *lme* function of the *nlme* package R (Pinheiro et al., 2021), with a fixed effect of time (BA) and treatment (CI) and their interaction (BA*CI), and site as the random factor. We validated all models by checking distributions of normality and equal variances, visually and statistically, using Levene's test homogeneity of variance and Shapiro–Wilk test to confirm normality of residuals. If normality and homoscedasticity were not met, data were log or sqrt transformed (Table S2). We used the R package “interplot” (Solt & Hu, 2021) to visualize the BACI model interaction terms.

We used non‐metric multidimensional scaling (nMDS) based on Bray–Curtis dissimilarity to assess variation in vegetation and ant species and functional group composition among treatments, as well as differences in soil physical and biochemical variables using Euclidian dissimilarities with the metaMDS function in the *vegan* package (Oksanen et al., 2007) in R. We applied a perMANOVA to the BACI model to test for multivariate differences in (a) soil physical, (b) soil biochemical, and (c) biotic variables (Table S1) using the “adonis” function in the vegan package in R.

## 3 RESULTS

### 3.1 Soil physical and biochemical responses

**FIGURE 3:**
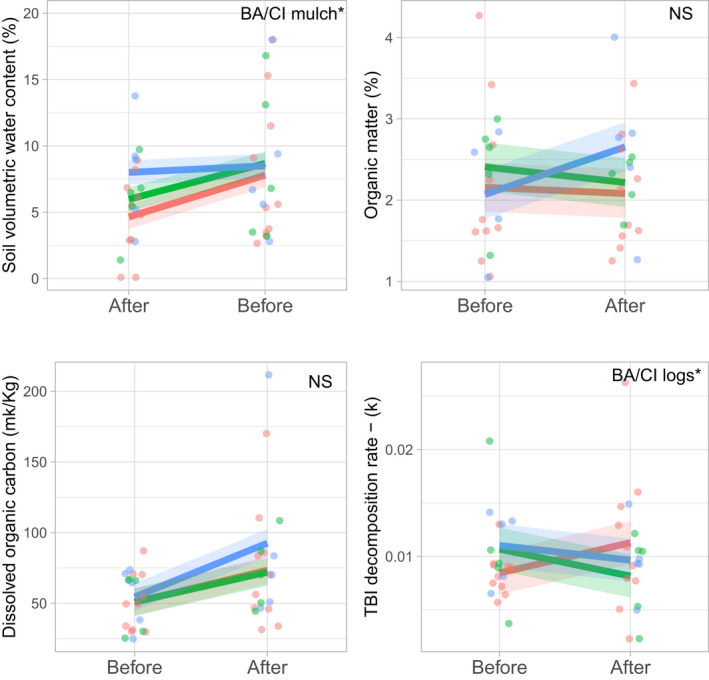
Mean effect of treatment (log [green] and mulch [blue] vs. control [red]) and time (before vs. 2 years after treatment application) on soil volumetric water content, organic matter, dissolved organic carbon, and decomposition rate (*n* = 5, ±1 SE)

Across all 14 soil biochemical and physical response variables, we found a significant BACI effect only for volumetric water content (*p* = .01) and decomposition (*p* = .03). In the mulch treatment, the BACI interaction effect showed that volumetric water content was significantly higher 2 years after application, compared to the control treatment plots (Table S2, Figure 3). In the log treatment, decomposition was significantly lower in the log treatment plots compared to the controls (Table S2, Figure 3). While we found statistically significant differences between periods for gravimetric water content, indicating a much drier sampling season in the late spring of 2019 (Figure S1), and also statistically significant differences between sites for dissolved organic nitrogen, nitrate, organic carbon, and microbial biomass nitrogen, the BACI interaction for those variables was not statistically significant (Table S2, Figures 3). While other soil properties (organic matter and dissolved organic carbon) showed a positive response to mulch addition, the effect was not statistically significant. We found no BACI treatment effect for multivariate differences for soil physical components (perMANOVA, BA:CI interaction, *p* = .87) as well as soil chemical components (perMANOVA, BA:CI interaction, *p* = .91).

While we measured penetration resistance across all plots using a handheld electronic penetrometer in 2017, almost all measurements in 2019 were error readings due to the extreme dryness of the soil. Therefore, we did not include penetration resistance data in the results section.

### 3.2 Herbaceous vegetation and ground cover

**FIGURE 4:**
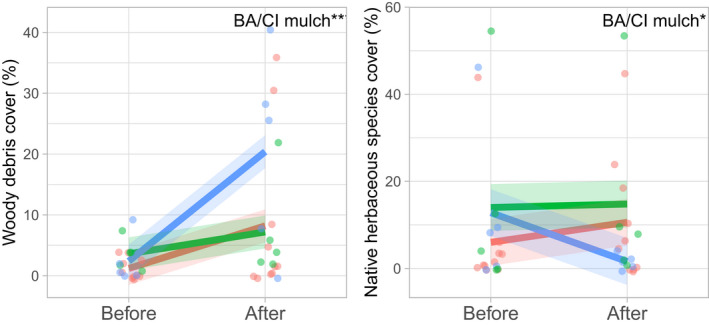
Mean effect of treatment (log [green] and mulch [blue] vs. control [red]) and time (before vs. 2 years after treatment application) on woody debris and native herbaceous species cover (*n* = 5, ±1 SE)

We recorded a total of 23 herbaceous plant species in the 2 sampling years across all plots, with equal proportions of native (52%) and exotic (48%) species. Average native species richness per plot was lower (5, ±0.8) compared to exotic species richness (7.2, ±1.2). The BACI interaction effect showed a significant decline in mean native species cover (*p =* .03*)* as well as a near significant decline in mean native species richness (*p* = .05) on the mulch‐treated sites, (Table S2, Figure 4). Treatments of mulch and logs had no effects on exotic species cover and richness or bare ground (Table S2, Figure 4). Woody debris cover in 2019 was significantly higher (*P* = .008) on plots with mulch‐added compared with control plots (Figure 4). Woody debris cover included any woody material smaller than 10 cm in diameter (twigs, small branches, and added mulch).

We did not find any distinct patterns of the BACI interaction in flora species composition using nMDS scaling (Figure S2). We found no BACI treatment effect for multivariate differences in herbaceous vegetation abundance (perMANOVA, BA:CI interaction, *p* = .99).

### 3.3 Ant community

We recorded a total of 83 species from 11 genera and 8 functional groups during the two sampling periods across all sites and treatments. The richest genera were *Melopherus* (21 species), *Camponotus* (16), *Iridomyrmex* (12), *Monomorium* (11), and *Pheidole* (8). The genus *Iridomyrmex* had by far the highest abundance of ants (78%), followed by *Melopherus* (8%), *Monomorium* (7%), *Pheidole* (2%), *Rhytidoponera* (2%), and *Camponotus* (1%). *Iridomyrmex chasei* was the most abundant species, contributing to two‐thirds (65%) of all captures. Across the eight functional groups, Dominant Dolichoderinae (*Iridomyrmex* spp.) were the most abundant (78%), followed by Hot Climate Specialists (15%). In contrast, species richness was highest for Hot Climate Specialists (45%), Subordinate Camponotini (19%), Dominant Dolichoderinae (14%), and Generalized Myrmicinae (10%). Four other functional groups were present in small numbers only and are therefore excluded from the results (Figure S3).

**FIGURE 5:**
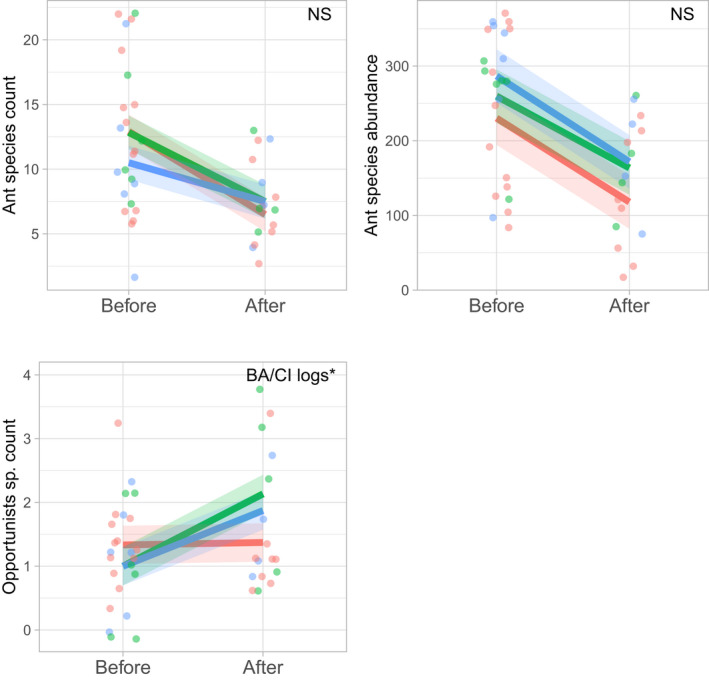
Mean effect of treatment (log [green] and mulch [blue] vs. control [red]) and time (before vs. 2 years after treatment application) on ant species richness and abundance, and species richness of opportunistic ants (*n* = 5, ±1 SE)

At the treatment level, overall species richness decreased across all control and treatment plots in 2019, but less so for the mulch‐treated plots and we detected a near‐significant BACI effect for overall species richness at the mulch treatment (*p* = .07). Species abundance also decreased across all control and treatment plots in 2019 and we detected no significant difference in species abundance (Table S2, Figure 5). For functional groups, we found a significant BACI effect for opportunistic ant species (*Rhytidoponera* spp.) showing a two‐fold increase in species richness at the log treatment plots (*p* = .03) (Table S2, Figure 5). Overall, *Rhytidoponera* species richness remained low on the log‐treated plots (mean = 2.2, range from 1 to 4). Generalized Myrmicinae showed an increase in mean abundance and richness at the mulch‐treated plots, however, as for the remaining functional groups, the difference was not statistically different (Table S2, Figure 5). We detected no distinct patterns of changes in ant communities between the BA and CI factors in the nMDS scaling plot (Figure S4). We found no BACI treatment effect for multivariate differences in ant species abundance (perMANOVA, BA:CI interaction, *p* = .99).

## 4 DISCUSSION

Our study showed that additional restoration actions on planted old fields can accelerate restoration outcomes to some extent. In particular, soil biophysical functions and effects on biodiversity showed promising, if few benefits of mulch and log additions. We interpret the limited responses to the semi‐arid climate, where abiotic and biotic ecosystem variables may respond only gradually over time or be linked to episodic climate events (e.g., Holmgren & Scheffer, 2001; Wainwright et al., 2012).

### 4.1 Soil biophysical function

In partial support of our first hypothesis, mulch addition to planted old fields increased woody debris cover and soil moisture, compared to control and log‐treated plots, although soil moisture was only higher in winter, but not in late spring. We also found increasing trends for organic matter and dissolved organic carbon. Higher soil water content and increasing trends for soil organic matter and dissolved organic carbon may be a first measurable signal of the vegetation–soil water feedback, indicating the restoration of ecosystem functions (Prober et al., 2014).

Increased soil moisture may be attributed to mulch providing a protective surface layer, similar to the effect of leaf litter (Sayer, 2006) and soil amendments such as biochar (Prober et al., 2014), therefore reducing water run‐off and delaying evaporation at the soil surface. However, soil moisture content measures in late spring did not show higher moisture content on the mulch‐treated plots, suggesting that mulch may only slow down the loss of top soil moisture content. Our predictions that woody debris additions would also reduce bulk density, increase soil carbon, soil microbial activity, and decomposition rates were not met.

Several factors may have contributed to not detecting further effects. Firstly, woody debris application rates were aligned with leaf litter and log biomass in mature reference vegetation systems. However, while woody debris in reference woodlands is continuously replenished, this is less so in young restoration sites as immature vegetation produces less plant litter. Furthermore, we found evidence that the mulch was patchy after 2 years, possibly due to redistribution of mulch by wind and fauna (Figure 2). Therefore, the physical barrier provided by the mulch may have been too shallow and patchy to provide an effective physical barrier for water retention even during the hotter season. A thicker mulch application may have also increased effects of soil organic matter and carbon, microbial biomass, and bulk density as shown in Biederman and Whisenant (2011).

Secondly, effects of indirect interactions following woody debris additions on the vegetation–soil water feedback such as increased soil organic matter and carbon, and therefore microbial activity and decomposition rates, may take longer time frames (i.e., 10+ years) to be detectable (Mao et al., 1992; Sayer, 2006). Furthermore, environmental conditions (temperature, soil moisture, and soil pH) influence microbial activity and decomposition rates, and therefore nutrient release, and in dry ecosystem such as ours the rate of detectable change is slow (Facelli & Pickett, 1991). Low decomposition rates are in line with other studies (Keuskamp et al., 2013; Ochoa‐Hueso et al., 2020), indicating that a 2‐year time frame was too short to show potential treatment outcomes. In more mesic systems, the addition of mulch has indicated recovery of several ecosystem functions such as increased soil moisture and decomposition rates (Dawes, 2010), as well as labile soil carbon, lower soil bulk density, and softer soil surface (Prober et al., 2014).

### 4.2 Biodiversity

The positive effects of mulch on soil physical functions were somewhat offset by a decline in native herbaceous species cover. Mulch may have posed a physical barrier to the germination of native and exotic herbaceous species (Beggy & Fehmi, 2016; Facelli, 1994; Xiong & Nilsson, 1999), as found by Prober et al. (2014) for small‐ but not large‐seeded native herbs. By contrast, a reduction in herbaceous exotic species cover and richness was not observed, consistent with findings by Prober et al. (2014). Although leaf litter can pose a physical barrier for plant seeds and reduce germination rate, more so for woody than herbaceous species (Facelli, 1994; Jiang et al., 2009), improved effects on soil biophysical condition (e.g., soil moisture, but also reduced bulk density, see Prober et al. (2014)) are beneficial to plant establishment.

The decline in native herbaceous species cover on the mulched plots was mainly driven by two species, each occurring at one individual plot only: *Dysphania melanocarpa* (J.M. Black) Mosyakin & Clemants and *Ptilotus polystachyus* (Gaudich.) F. Muell. Both species are not diminutive. *D. melanocarpa* is a medium tall herb, and *P. polystachyus* can grow over 1 m tall, with a seed size of 2–3 mm (Western Australian Herbarium, 1997). In fact, the latter is a common and widespread native herb (Fensham et al., 2011), well adapted to low and high soil phosphorus environments (Hammer et al., 2020; Ryan et al., 2009) and often found in high abundance on disturbed post‐agricultural land. *Ptilotus polystachyus* has also been observed to grow well and outcompete *Lupinus cosentinii*, a grain legume, following substantial summer rains, but less so during years with low summer rain (B. Parkhurst, pers. com.). This observation is in line with our recorded high cover of *P. polystachyus* in 2017 coinciding with high summer rains, and its absence in 2019, which had very little summer rain (Figure S1). While this observation requires hypothesis‐driven testing, it is important to consider idiosyncratic effects of climate and species‐specific responses and competitive interactions in the study region (Dwyer et al., 2015), when interpreting the effectiveness of mulch as a restoration tool.

Significant treatment effects were only observed for opportunistic ants. These responded positively to the addition of logs, and to some extend mulch, with an increase in species richness (*Rhytidoponera* spp.*)*. Opportunists, especially *Rhytidoponera* spp., favor habitats that support low ant diversity and increase in abundance following habitat disturbance (e.g., fire and mining [Andersen, 2019; Hoffmann & Andersen, 2003]). However, increased species richness patterns of opportunistic ants were only driven by a few species of *Rhytidoponea* spp., and this trend may therefore not reflect broader changes.

While we had hypothesized that mulch and log additions would increase abundance and diversity of functional groups that forage and nest in woody debris, this was not the case, possibly due to unsuitable log habitat quality. Saproxylic invertebrate, including ants, respond strongly to not only macrohabitat quality surrounding the log (i.e., land use type) but also microhabitat features (i.e., decay state, humidity, leaf litter, and canopy cover) directly associated with logs (Gibb et al., 2006). The logs we applied were not decayed and are therefore less favored as nesting sites by ant species as opposed to rotten logs (Gibb et al., 2012). Therefore, more highly decayed logs, placed under tree canopy, may have been more suitable to accelerate restoration outcomes for some ant species, as has been shown for other invertebrate groups (e.g., saproxylic beetles in Sandström et al., 2019). However, studies on the responses of ants to woody debris addition and required habitat quality are very rare, even more so in a restoration context, therefore require further investigation (Sandström et al., 2019; Seibold et al., 2015).

## 5 CONCLUSION

This MBACI experiment has indicated desirable effects of woody debris additions on soil moisture and ant communities, but overall evidence that woody debris additions are a suitable restoration approach to accelerate restoration outcomes on old fields in agricultural landscapes remains inconclusive. Further research is needed to determine whether the type, quantity, and context of mulch and log additions can improve their effectiveness for old field restoration in semi‐arid regions, in particular for soil physical and biochemical functions, without negative effects on biodiversity. In addition, the feasibility of woody debris additions as a restoration tool for restoration practitioners without exhausting logistical and financial resources needs to be examined.

## AUTHOR CONTRIBUTIONS

**Tina Parkhurst:** Conceptualization (equal); data curation (lead); formal analysis (lead); investigation (lead); methodology (equal); project administration (lead); visualization (lead); writing – original draft (lead); writing – review and editing (equal). **Suzanne Prober:** Conceptualization (equal); formal analysis (equal); funding acquisition (equal); methodology (equal); supervision (equal); visualization (equal); writing – original draft (supporting); writing – review and editing (equal). **Mark Farrell:** Conceptualization (equal); formal analysis (equal); investigation (equal); methodology (equal); resources (equal); supervision (equal); writing – original draft (equal); writing – review and editing (equal). **Rachel Jayne Standish:** Conceptualization (equal); formal analysis (equal); funding acquisition (equal); investigation (equal); methodology (equal); supervision (equal); visualization (equal); writing – review and editing (equal).

## CONFLICT OF INTEREST

The authors declare no conflict of interest.

## Supporting information


Appendix S1

## Data Availability

Data available via the Terrestrial Ecosystem Research Network's Data Discovery Portal, https://doi.org/10.25901/q2sc‐w119.
