# Supplementary material for: Abiotic and biotic responses to woody debris additions in restored old fields in a multi‐site Before‐After‐Control‐Impact experiment
Source: Ecol Evol. 2022 Jul 4;12(7):e9058. doi: 10.1002/ece3.9058 (PMC9251846; doi:10.1002/ece3.9058)
Supplement: Supplementary file 1 — Appendix S1 [file ECE3-12-e9058-s001.docx]

# Supporting Information

## Abiotic and biotic responses to woody debris additions in restored old fields in a multi-site Before-After-Control-Impact experiment

Tina Parkhurst^1,2^, Suzanne M Prober^2,4^, Mark Farrell^3^, Rachel J Standish^1^

^1^Harry Butler Institute, Murdoch University, Murdoch, Western Australia, 6150

^2^CSIRO Land and Water, Wembley, Western Australia, 6913

^3^CSIRO Agriculture and Food, Glen Osmond, South Australia, 5064

^4^School of Biological Sciences, The University of Western Australia, Crawley, Western Australia, 6009

Correspondence: Tina Parkhurst, [tina.parkhurst@murdoch.edu.au](mailto:tina.parkhurst@murdoch.edu.au)

## Detailed sampling methods

Table S1. MBACI Response variables and sampling details

| Variable | | Date - Before | Date - After | No of Sites | Plot replicates |
| --- | --- | --- | --- | --- | --- |
| Soil physical properties | | | | | |
| Bulk Density | | September 2017 | September 2019 | 5 | 3 |
| Gravimetric Water Content | | September 2017 | September 2019 | 5 | 3 |
| Penetration resistance | | November 2017 | November 2019 | 5 | 10 (2017 only) |
| Volumetric Water Content | | August 2017 | August 2019 | 5 | 10 |
| Soil biochemical properties | | | | | |
| Dissolved organic carbon | | November 2017 | November 2019 | 5 | 10 |
| Dissolved organic nitrogen | | November 2017 | November 2019 | 5 | 10 |
| Dissolved inorganic nitrogen (Nitrate) | | November 2017 | November 2019 | 5 | 10 |
| Dissolved inorganic nitrogen (Ammonium) | | November 2017 | November 2019 | 5 | 10 |
| Free amino acids - FAA | | November 2017 | November 2019 | 5 | 10 |
| Organic Carbon % | | November 2017 | November 2019 | 5 | 10 |
| Organic Matter % | | November 2017 | November 2019 | 5 | 10 |
| Microbial biomass carbon | | November 2017 | November 2019 | 5 | 10 |
| Microbial biomass nitrogen | | November 2017 | November 2019 | 5 | 10 |
| Tea Bag Index – decomposition rate (k) | | August to November 2017 | August to November 2019 | 5 | 3 |
| Tea Bag Index –stabilisation factor (S) | | August to November 2017 | August to November 2019 | 5 | 3 |
| Herbaceous vegetation | | | | | |
| Exotic species cover (abundance) | | October 2017 | September 2019 | 5 | 50 |
| Exotic species richness | | October 2017 | September 2019 | 5 | 50 |
| Native species cover (abundance) | | October 2017 | September 2019 | 5 | 50 |
| Native species richness | | October 2017 | September 2019 | 5 | 50 |
| Bare ground | | October 2017 | September 2019 | 5 | 50 |
| Woody debris | | October 2017 | September 2019 | 5 | 50 |
| Ant assemblages | | | | | |
| Species abundance | September 2017 | | September 2017 | 5 | 5 |
| Species richness | September 2017 | | September 2017 | 5 | 5 |
| Dominant Dolichoderinae abundance | September 2017 | | September 2017 | 5 | 5 |
| Dominant Dolichoderinae richness | September 2017 | | September 2017 | 5 | 5 |
| Generalised Myrmicinae abundance | September 2017 | | September 2017 | 5 | 5 |
| Generalised Myrmicinae richness | September 2017 | | September 2017 | 5 | 5 |
| Hot Climate Specialists abundance | September 2017 | | September 2017 | 5 | 5 |
| Hot Climate Specialists richness | September 2017 | | September 2017 | 5 | 5 |
| Opportunists abundance | September 2017 | | September 2017 | 5 | 5 |
| Opportunists richness | September 2017 | | September 2017 | 5 | 5 |

Fig S1. Rainfall graph for the 2 sampling years.

### Soil sampling

#### Volumetric Water Content

We measured volumetric topsoil water content (12 cm) at 10 random points in each plot using a hand-held soil moisture probe. To minimize variation of soil moisture due to time of day, soil moisture for each plot was measured during the early morning hours.

#### Bulk Density and Gravimetric Water Content

At each plot, three soil bulk density samples of the 0-10 cm topsoil were taken in a steel ring following the TERN AusPlots method (White, Sparrow et al. 2012). Samples were placed in plastic bags and sealed for transport. At the laboratory, soil bulk density samples were then weighed and transferred to paper bags for oven drying (48 hours at 105 °C). The dry weight was measured to three decimal points. Bulk density was calculated by dividing the mass of the oven dried soil (g) by the volume of the ring (cm^3^). Gravimetric water content of the soil was determined by subtracting the weight of the dry soil from the weight of the moist soil, and then dividing by the weight of the dry soil.

***Penetration resistance***

We measured penetration resistance of the soil in 2017 at 10 random points across the 5 x 5 m plot using a penetrologger with a 1 cm^2^ - and a 60° - top angle cone (Eijkelkamp Agrisearch Equipment, Giesbeek, the Netherlands). The device was pushed vertically into the soil at a constant speed of 2cm/s. The resistance to penetration was stored as force (Newton), in the data logger and electronically transferred to a spreadsheet.

#### Organic Carbon, Organic Matter, Organic Moisture

Soil samples to 10 cm depth were collected at 10 points across each plot. The 10 samples were randomly collected and then combined. Sampling occurred in November 2017 and again in November 2019. Samples were stored at 4 °C in plastic zip-lock bags until delivery to the CSBP Limited (Bibra Lake, Western Australia) laboratories. Soil samples were dried at 80 °C and ground and sieved to 2 mm. Organic carbon was measured using the Walkley and Black method, 6A1, and Total Organic Matter (Loss on Ignition) and Organic Moisture) were measured using the Rayment and Lyons Method 6G1.

***Dissolved nitrogen and carbon pools***

Dissolved nitrogen and carbon were extracted from the soil using a 1:5 soil:solution 0.5 M K_2_SO_4_ extract, shaken for 30 minutes before centrifugation and filtration. Given the dynamic nature of these pools, soil samples were frozen upon collection in the field using a portable fridge/freezer. After extraction, extracts were again immediately frozen until analysis. Salt solutions such as K_2_SO_4_ are typically preferred over water as they can extract a more representative sample of bound substrates such as ammonium, and the high salt content acts as an inhibitor of microbial activity in the short term during the extraction process.

***Dissolved inorganic nitrogen – NO_3_ & NH_4_***

Nitrate and ammonium were quantified on the K_2_SO_4_ extracts using colourimetric approaches on the same multimode plate reader as for the phosphorus analysis following Miranda and others (2001) and Mulvaney (1996), respectively. The nitrate analysis also captures any nitrite (NO_2_^-^), though it is usually only found in trace amounts due to its rapid transformation to nitrate.

***Free amino acids - FAA***

Free amino acids were quantified on the K_2_SO_4_ extracts using a fluorimetric approach with *o*-phthaldialdehyde and *b*-mercaptoethanol in a borate buffer at pH 9.5 (Jones and others, 2002), followed by analysis on the same multimode plate reader.

***Dissolved organic carbon and nitrogen – DOC and DON (DTN –sum (NH_4_+NO_3_))***

There is no direct analysis of bulk DON (unlike target N species such as FAAs), rather this value is calculated by subtracting the sum of DIN from a total dissolved nitrogen (TDN) measurement. For DOC, this value can either be derived by subtraction of a dissolved inorganic carbon (DOC) value from a total dissolved carbon (TDC) value, or through the direct analysis of non-purgeable organic carbon (NPOC) after pre-treatment of the sample with HCl and agitation by vortexing or purging with a stream of He. In this study, we quantified DOC and TDN by high temperature combustion (Thermalox TOC/N Analayser; Analytical Sciences Ltd., Tewkesbury, UK), with DON being derived after subtraction of DIN from the TDN value.

***Microbial biomass carbon and nitrogen (MBC, MBN)***

Microbial carbon and nitrogen pools were estimated using the chloroform fumigation extraction (CFE) methods of Vance and others (1987). The principle behind this is that microbial lipid cell membranes are lysed in a chloroform atmosphere, releasing the carbon and nitrogen they contain into an extractable form. The concentrations of dissolved carbon and nitrogen in this extract are then quantified as above for DOC and TDN, before subtraction of the unfumigated value for the same sample, and the application of a correction factor (K_ec_ for carbon, K_en_for nitrogen) to account for carbon and nitrogen contained in the lysed microbial cells that was not extracted (Voroney and others, 2008). In this project, we quantified microbial biomass carbon (MBC) and microbial biomass nitrogen (MBN) as per these methods.

### Decomposition

Five green and roiboos teabags were buried at each plot in August 2017 and again in August 2019, following the standardized Tea Bag Index (TBI) method developed by Keuskamp, Dingemans, Lehtinen, Sarneel and Hefting (2013). After 3 months, in November 2017 and 2019, teabags were retrieved, and stored in paper bags for transport to the laboratory. After drying teabags for 48 hours at 90 °C, the dry weight without cord and label was recorded to three decimal points.

### Vegetation sampling

All annual and perennial plant species were recorded in spring 2017 within each plot and identified to genus and species level where possible. Nomenclatures follow the (Western Australian Herbarium 1997-). A point intercept method (Prober, Thiele, Lunt & Koen 2005) was used to quantify cover of individual species, total vegetation cover and substrate types (i.e., bare ground, litter cover, plant cover). Ground cover, individual species intercepting at every 0.5m along five parallel, evenly spaced 5 m transects across each plot were recorded using a vertically placed dowel (8mm wide, 2m tall), resulting in 50 intercepting points per plot. To calculate species richness and cover across different life history and growth forms, species were classified into the following groups: total, native grasses, native perennial forbs, native annual forbs, exotic grasses and exotic annual forbs using the Western Australian Herbarium (2017) classification.

### Ant sampling

Ants were surveyed using pitfall traps, which provide reliable information on ant species richness and relative abundance in open habitats (Andersen 1991). Five pitfall traps (4.5 cm diameter) were established at each plot in September 2017 and again in September 2019. Traps were left closed for one week to minimise the digging-in effect on sampling. After one week, lids were removed and traps half filled with a 50% propylene glycol solution. All traps were collected after five days. After collection, catches were washed and stored in 70% ethanol. All ants were sorted to species. All ant species were classified into functional groups as proposed by Andersen (1995). Voucher specimens for all ant species are stored at the Commonwealth Scientific and Industrial Research Laboratory in Darwin, Northern Territory, Australia. Abundance of each species per trap was capped at 50 individuals to prevent data distortions from traps that were potentially placed near nests or foraging trails. Species abundances in individual traps were summed to obtain total abundances per species per plot.

Table S2. BACI analysis with the parameter estimates and significance tests from linear mixed-effects models fitted to response variables (* indicates data transformed prior to analysis).

| Response variable | Model variable | Coefficient | | SE | *P* | |
| --- | --- | --- | --- | --- | --- | --- |
| *Experimental mulch and log additions* | | | | | | |
| Fine and coarse woody debris  (mulch and log additions) | Intercept  Before vs After = *BA*  Control vs Impact ‘*logs’* = *CI(L)*  Control vs Impact ‘*mulch’* = *CI(M)*  *BA*CI(L)*  *BA*CI(M)* | 4.7  4.95  0.7  6.7  -2.4  7.78 | | 3.06  3.76  1.92  1.92  2.71  2.71 | 0.14  0.25  0.72  ***0.002***  0.38  ***0.008*** | |
| *Soil physical properties* | | | | | | |
| Bulk density | Intercept  Before vs After = *BA*  Control vs Impact ‘*logs’* = *CI(L)*  Control vs Impact ‘*mulch’* = *CI(M)*  *BA*CI(L)*  *BA*CI(M)* | 1.51  -0.06  -0.02  0.02  0.03  0.06 | | 0.04  0.05  0.03  0.03  0.05  0.05 | ***0.000***  0.34  0.57  0.55  0.47  0.22 | |
| Gravimetric water content*  (sqrt transformed) | Intercept  Before vs After = *BA*  Control vs Impact ‘*logs’* = *CI(L)*  Control vs Impact ‘*mulch’* = *CI(M)*  *BA*CI(L)*  *BA*CI(M)* | 0.84  -0.72  -0.003  0.005  -0.04  -0.009 | | 0.11  0.16  0.05  0.05  0.07  0.07 | ***0.000***  ***0.044***  0.95  0.92  0.58  0.89 | |
| Volumetric water content | Intercept  Before vs After = *BA*  Control vs Impact ‘*logs’* = *CI(L)*  Control vs Impact ‘*mulch’* = *CI(M)*  *BA*CI(L)*  *BA*CI(M)* | 6.22  -2.22  1.11  2.03  0.31  1.87 | | 1.85  1.46  0.49  0.49  0.69  0.69 | ***0.002***  0.201  ***0.031***  ***0.0003***  0.656  ***0.011*** | |
| *Soil biochemical properties* | | | | | | |
| Dissolved organic carbon*  (sqrt transf) | Intercept  Before vs After = *BA*  Control vs Impact ‘*logs’* = *CI(L)*  Control vs Impact ‘*mulch’* = *CI(M)*  *BA*CI(L)*  *BA*CI(M)* | 7.6  0.9  0.4  0.6  0.6  0.5 | 0.74  0.66  0.33  0.33  0.46  0.46 | | ***<0.001***  0.34  0.89  0.09  0.89  0.32 | |
| Dissolved organic nitrogen* (log transf) | Intercept  Before vs After = *BA*  Control vs Impact ‘*logs’* = *CI(L)*  Control vs Impact ‘*mulch’* = *CI(M)*  *BA*CI(L)*  *BA*CI(M)* | 1.81  0.59  -0.02  0.05  0.12  -0.14 | 0.16  0.22  0.11  0.11  0.16  0.16 | | ***<0.001***  ***0.05***  0.88  0.65  0.46  0.38 | |
| Dissolved inorganic Nitrogen (Nitrate, NO_3_)*  (log transformed) | Intercept  Before vs After = *BA*  Control vs Impact ‘*logs’* = *CI(L)*  Control vs Impact ‘*mulch’* = *CI(M)*  *BA*CI(L)*  *BA*CI(M)* | 1.52  -0.50  0.01  -0.003  0.16  0.09 | 0.39  0.16  0.14  0.14  0.19  0.19 | | ***<0.001***  ***<0.001***  0.75  0.98  0.36  0.81 | |
| Dissolved inorganic Nitrogen (Ammonium, NH_4_)* (log transformed) | Intercept  Before vs After = *BA*  Control vs Impact ‘*logs’* = *CI(L)*  Control vs Impact ‘*mulch’* = *CI(M)*  *BA*CI(L)*  *BA*CI(M)* | 1.25  0.41  0.07  0.15  0.03  0.16 | 0.28  030  0.40  0.40  0.44  0.44 | | ***<0.001***  *0.06*  0.21  0.86  0.72  0.95 | |
| Free amino acids*  (log transformed) | Intercept  Before vs After = *BA*  Control vs Impact ‘*logs’* = *CI(L)*  Control vs Impact ‘*mulch’* = *CI(M)*  *BA*CI(L)*  *BA*CI(M)* | -0.35  0.17  0.02  0.02  0.07  0.07 | 0.08  0.11  0.04  0.04  0.06  0.06 | | ***<0.001***  0.18  0.63  0.71  0.28  0.25 | |
| Organic Carbon*  (log transformed) | Intercept  Before vs After = *BA*  Control vs Impact ‘*logs’* = *CI(L)*  Control vs Impact ‘*mulch’* = *CI(M)*  *BA*CI(L)*  *BA*CI(M)* | -0.13  -0.06  0.02  0.06  0.02  0.03 | 0.06  0.03  0.03  0.03  0.04  0.04 | | 0.05  0.08  0.63  ***0.04***  0.68  0.54 | |
| Organic matter*  (sqrt transformed) | Intercept  Before vs After = *BA*  Control vs Impact ‘*logs’* = *CI(L)*  Control vs Impact ‘*mulch’* = *CI(M)*  *BA*CI(L)*  *BA*CI(M)* | 1.43  -0.007  0.08  0.08  -0.03  0.13 | 0.09  0.06  0.06  0.06  0.09  0.09 | | ***<0.001***  0.91  0.23  0.23  0.75  0.15 | |
| Microbial biomass carbon | Intercept  Before vs After = *BA*  Control vs Impact ‘*logs’* = *CI(L)*  Control vs Impact ‘*mulch’* = *CI(M)*  *BA*CI(L)*  *BA*CI(M)* | 215.5  -28.85  27.14  75.18  -34.04  5.46 | 49.71  26.44  32.44  32.44  45.87  45.87 | | ***<0.001***  0.34  0.41  ***0.03***  0.46  0.91 | |
| Microbial biomass nitrogen* (sqrt transf) | Intercept  Before vs After = *BA*  Control vs Impact ‘*logs’* = *CI(L)*  Control vs Impact ‘*mulch’* = *CI(M)*  *BA*CI(L)*  *BA*CI(M)* | 3.79  -1.35  0.11  0.94  -0.23  0.63 | 0.63  0.45  0.38  0.38  0.54  0.54 | | ***<0.001***  ***0.04***  0.78  ***0.02***  0.67  0.25 | |
| TBI - Decomposition rate (k)* (sqrt transf) | Intercept  Before vs After = *BA*  Control vs Impact ‘*logs’* = *CI(L)*  Control vs Impact ‘*mulch’* = *CI(M)*  *BA*CI(L)*  *BA*CI(M)* | 0.09  0.01  -0.003  0.004  -0.02  -0.01 | 0.01  0.01  0.01  0.01  0.01  0.01 | | ***<0.001***  0.46  0.53  0.45  ***0.03***  *0.08* | |
| TBI – Litter stabilisation factor (S) | Intercept  Before vs After = *BA*  Control vs Impact ‘*logs’* = *CI(L)*  Control vs Impact ‘*mulch’* = *CI(M)*  *BA*CI(L)*  *BA*CI(M)* | 0.42  0.05  -0.004  0.006  -0.01  0.00 | 0.02  0.02  0.02  0.02  0.03  0.03 | | ***<0.001***  *0.08*  0.79  0.74  0.68  0.98 | |
| *Herbaceous vegetation* | | | | | | |
| Exotic species richness | Intercept  Before vs After = *BA*  Control vs Impact ‘*logs’* = *CI(L)*  Control vs Impact ‘*mulch’* = *CI(M)*  *BA*CI(L)*  *BA*CI(M* | 2.45  0.64  -0.15  0.25  0.64  -0.49 | | 0.45  0.35  0.32  0.32  0.45  0.45 | ***<0.001***  0.14  0.64  0.44  0.17  0.28 | |
| Exotic species relative abundance | Intercept  Before vs After = *BA*  Control vs Impact ‘*logs’* = *CI(L)*  Control vs Impact ‘*mulch’* = *CI(M)*  *BA*CI(L)*  *BA*CI(M)* | 29.3  15.41  1.7  12.7  1.27  -1.84 | | 9.39  9.2  3.69  3.69  5.22  5.22 | ***<0.01***  0.17  0.64  ***<0.01***  0.81  0.73 | |
| Native species richness | Intercept  Before vs After = *BA*  Control vs Impact ‘*logs’* = *CI(L)*  Control vs Impact ‘*mulch’* = *CI(M)*  *BA*CI(L)*  *BA*CI(M)* | 0.85  0.35  0.35  -0.05  -0.35  -0.34 | | 0.27  0.37  0.24  0.24  0.34  0.34 | ***<0.01***  0.39  0.16  0.84  0.32  ***0.05*** | |
| Native species cover | Intercept  Before vs After = *BA*  Control vs Impact ‘*logs’* = *CI(L)*  Control vs Impact ‘*mulch’* = *CI(M)*  *BA*CI(L)*  *BA*CI(M)* | 8.3  3.25  6.10  -1.10  -2.69  -11.17 | | 4.78  6.77  3.79  3.79  5.37  5.37 | 0.09  0.65  0.12  0.77  0.62  ***0.04*** | |
| Bare ground | Intercept  Before vs After = *BA*  Control vs Impact ‘*logs’* = *CI(L)*  Control vs Impact ‘*mulch’* = *CI(M)*  *BA*CI(L)*  *BA*CI(M)* | 37.6  -11.88  -9.2  -17.4  1.69  2.54 | | 8.88  4.21  4.34  4.34  6.14  6.14 | ***<0.001***  *0.05*  ***0.04***  ***<0.001***  0.78  0.68 | |
| *Ant assemblages* | | | | | | |
| Species abundance | Intercept  Before vs After = *BA*  Control vs Impact ‘*logs’* = *CI(L)*  Control vs Impact ‘*mulch’* = *CI(M)*  *BA*CI(L)*  *BA*CI(M)* | 174.12  -79.38  37.53  55.15  11.46  -1.79 | | 28.21  35.68  25.28  25.28  35.75  35.75 | | ***<0.001***  0.11  0.15  ***0.03***  0.75  0.96 |
| Species richness | Intercept  Before vs After = *BA*  Control vs Impact ‘*logs’* = *CI(L)*  Control vs Impact ‘*mulch’* = *CI(M)*  *BA*CI(L)*  *BA*CI(M)* | 9.7  -4.54  0.45  0.71  0.77  2.42 | | 1.83  1.5  0.91  0.91  1.29  1.29 | | ***<0.001***  ***0.05***  0.62  0.44  0.56  *0.07* |
| Dominant Dolichoderinae  abundance | Intercept  Before vs After = *BA*  Control vs Impact ‘*logs’* = *CI(L)*  Control vs Impact ‘*mulch’* = *CI(M)*  *BA*CI(L)*  *BA*CI(M)* | 117.39  -72.87  43.08  67.21  11.78  -8.78 | | 32.29  22.14  18.56  18.94  26.24  26.78 | | ***<0.01***  ***0.03***  ***0.03***  ***<0.01***  0.65  0.75 |
| Dominant Dolichoderinae  richness | Intercept  Before vs After = *BA*  Control vs Impact ‘*logs’* = *CI(L)*  Control vs Impact ‘*mulch’* = *CI(M)*  *BA*CI(L)*  *BA*CI(M)* | 2.43  -0.56  0.34  0.15  0.25  0.68 | | 0.36  0.37  0.27  0.27  0.38  0.38 | | ***<0.001***  0.20  0.21  0.59  0.52  0.09 |
| Generalised Myrmicinae  abundance* (sqrt transformed) | Intercept  Before vs After = *BA*  Control vs Impact ‘*logs’* = *CI(L)*  Control vs Impact ‘*mulch’* = *CI(M)*  *BA*CI(L)*  *BA*CI(M)* | 1.47  -0.77  0.32  0.54  -0.36  0.76 | | 0.43  0.45  0.52  0.54  0.73  0.76 | | ***<0.01***  0.16  0.53  0.32  0.62  0.32 |
| Generalised Myrmicinae  richness | Intercept  Before vs After = *BA*  Control vs Impact ‘*logs’* = *CI(L)*  Control vs Impact ‘*mulch’* = *CI(M)*  *BA*CI(L)*  *BA*CI(M)* | 0.83  -0.23  0.16  0.19  0.01  0.26 | | 0.20  0.23  0.26  0.27  0.36  0.37 | | ***<0.001***  0.36  0.54  0.48  0.97  0.49 |
| Hot Climate Specialists abundance*  (log transform) | Intercept  Before vs After = *BA*  Control vs Impact ‘*logs’* = *CI(L)*  Control vs Impact ‘*mulch’* = *CI(M)*  *BA*CI(L)*  *BA*CI(M)* | 2.71  -1.25  0.30  -0.08  0.57  0.55 | | 0.33  0.32  0.31  0.32  0.44  0.45 | | ***<0.001***  ***0.02***  0.34  0.08  0.21  0.24 |
| Hot Climate Specialists richness | Intercept  Before vs After = *BA*  Control vs Impact ‘*logs’* = *CI(L)*  Control vs Impact ‘*mulch’* = *CI(M)*  *BA*CI(L)*  *BA*CI(M)* | 3.88  -3.12  -0.28  -1.0  0.43  1.06 | | 0.67  0.77  0.61  0.63  0.87  0.89 | | ***<0.001***  ***0.02***  0.65  0.12  0.63  0.24 |
| Opportunists abundance | Intercept  Before vs After = *BA*  Control vs Impact ‘*logs’* = *CI(L)*  Control vs Impact ‘*mulch’* = *CI(M)*  *BA*CI(L)*  *BA*CI(M)* | 4.35  -0.57  0.79  1.42  0.77  -0.24 | | 1.46  1.94  1.81  1.87  2.56  2.64 | | ***<0.01***  0.78  0.66  0.45  0.76  0.93 |
| Opportunists richness | Intercept  Before vs After = *BA*  Control vs Impact ‘*logs’* = *CI(L)*  Control vs Impact ‘*mulch’* = *CI(M)*  *BA*CI(L)*  *BA*CI(M)* | 1.35  0.03  0.21  0.08  0.77  0.59 | | 0.28  0.31  0.25  0.26  0.36  0.37 | | ***<0.001***  0.93  0.41  0.75  ***0.04***  0.12 |


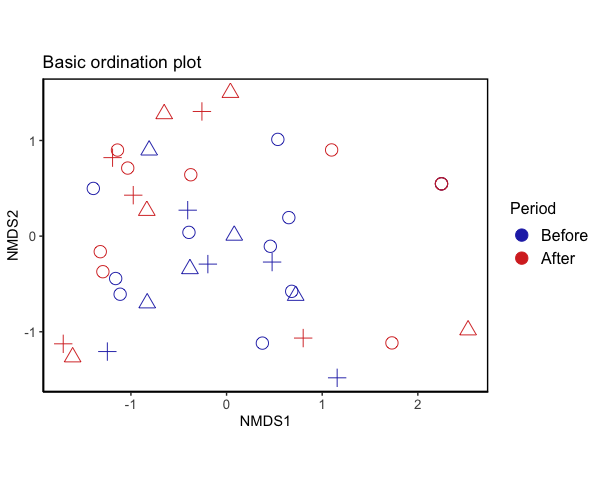


Fig S2: NMDS of herbaceous flora abundance data. Circles= controls, triangles = mulch, plus = logs.

Fig S3. Proportional abundance of ant functional groups across MBACI treatments.


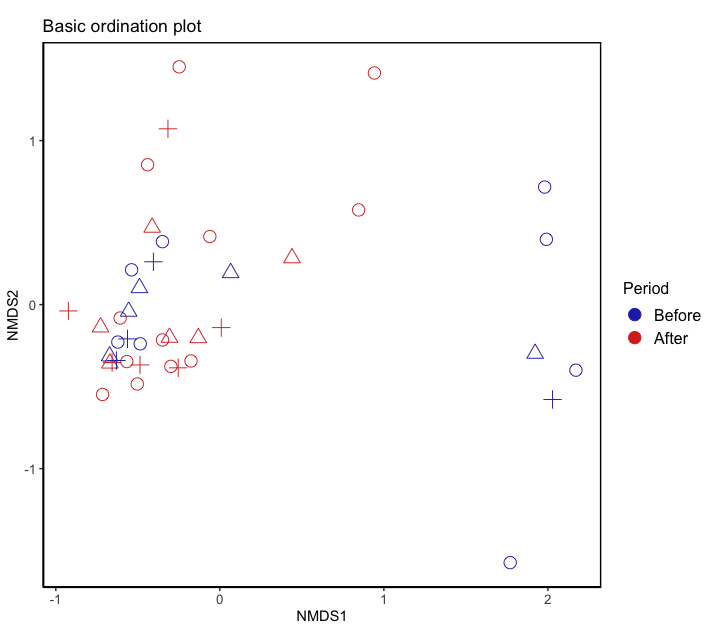


Fig S4. NMDS of ant abundance data. Circles= controls, triangles = mulch, plus = logs.

## References

Andersen, A 1991, 'Sampling communities of ground‐foraging ants: pitfall catches compared with quadrat counts in an Australian tropical savanna', Australian Journal of Ecology, vol. 16, no. 3, pp. 273-279.

Andersen, A 1995, 'A classification of Australian ant communities, based on functional groups which parallel plant life-forms in relation to stress and disturbance', Journal of Biogeography, pp. 15-29.

Keuskamp, JA, Dingemans, BJJ, Lehtinen, T, Sarneel, JM & Hefting, MM 2013, 'Tea Bag Index: A novel approach to collect uniform decomposition data across ecosystems', Methods in Ecology and Evolution, vol. 4, no. 11, pp. 1070-1075. doi: 10.1111/2041-210X.12097.

Prober, S, Thiele, K, Lunt, I & Koen, T 2005, 'Restoring ecological function in temperate grassy woodlands: manipulating soil nutrients, exotic annuals and native perennial grasses through carbon supplements and spring burns', Journal of Applied Ecology, vol. 42, no. 6, pp. 1073-1085.

Western Australian Herbarium 1997-, *FloraBase—the Western Australian Flora*, Department of Biodiversity, Conservation and Attractions, Perth, Western Australia, viewed July 2021, <<https://florabase.dpaw.wa.gov.au/>>.

White, A, et al. 2012, *AusPlots Rangelands Survey Protocols Manual, Version 1.2. 9 2012*, University of Adelaide Press, South Australia, www. tern. org. au/rs/7/sites/998/user_uploads/File/AusPlots% 20Rangelands% 20manual% 20versions/AusPlots% 20Rangelands% 20Survey% 20Protocols% 20Manual% 20v1.
